# Influence of playing surface on match injury risk in men's professional rugby union in England (2013–2019)

**DOI:** 10.1111/sms.14226

**Published:** 2022-09-04

**Authors:** Charlotte M. Robertson, Sean Williams, Stephen W. West, Lindsay Starling, Simon Kemp, Matt Cross, Keith A. Stokes

**Affiliations:** ^1^ Centre for Health and Injury and Illness Prevention in Sport, Department for Health University of Bath Bath UK; ^2^ Sport Injury Prevention Research Centre, Faculty of Kinesiology, University of Calgary Calgary Canada; ^3^ O'Brien Institute for Public Health University of Calgary Calgary Canada; ^4^ Rugby Football Union Twickenham UK; ^5^ London School of Hygiene and Tropical Medicine London UK; ^6^ Premiership Rugby Twickenham UK

**Keywords:** artificial surface, concussion, hybrid surface, injury, natural grass, pitch surface, rugby union

## Abstract

The use of artificial playing surfaces in professional rugby union is growing, but their effect on the injury risk profile remains unclear. The aim of this study was to examine the effect of playing surface on match injury risk in men's professional rugby in England. Six seasons of injury data (2013/14–2018/19) were collected from 15 professional English, men's rugby teams participating in domestic and European competition. The incidence, severity, and burden of match injuries were compared across playing surfaces. The dataset included 3351 injuries from a combined European and domestic data set (separated in to 2 categories; artificial and natural/hybrid surfaces) and 2675 injuries from a domestic only dataset (separated into 3 categories; artificial, hybrid, and natural surfaces). There were no differences in incidence rates between surface types on combined European and domestic match data, but injury burden was significantly greater on artificial (3082 days/1000 h, 95% CI 2847‐3337) in comparison with natural/hybrid surfaces (2364 days/1000 h, 95% CI 2277–2454, *p* < 0.001). These differences were primarily driven by a significantly greater mean severity of hip/groin, and foot/toe injuries on artificial surfaces. This is the largest study to date to examine the relationship between surface type and injury risk in rugby union. The average severity and burden of injuries sustained on artificial surfaces was significantly greater compared with those sustained on hybrid/natural grass surfaces. This study can inform those involved in selection of surface for elite sport, weighing up the positive and negative elements of the varying surface types.

## 1 INTRODUCTION

The playing surfaces used by top tier, English Rugby Union (herein referred to as “rugby”) teams fit into three broad categories: artificial, hybrid, and natural grass. Hybrid playing surfaces are made of predominantly natural grass but also contain synthetic material in various forms to aid in the stability and longevity of the surface. Playing surfaces containing artificial fibers have a number of benefits over natural grass surfaces, including being easier to maintain, having greater use‐tolerance, and being more resistant to broader weather changes.^1^ The first fully artificial match play surface in English elite domestic competition was introduced in the 2013/14 season, with three more Premiership teams installing fully artificial surfaces in 2014/15, 2016/17, and 2021/22.

Rugby is a field‐based, invasion, team sport.^2^ When comparing rugby in this population with other team sports, both the incidence and severity of injury are relatively high (87/1000 h and 25 days per injury,^3^ respectively). Previous research comparing injuries in matches played on artificial and natural grass surfaces in rugby has reported inconsistent overall injury incidence rates and severities between playing surfaces.^4^, ^5^, ^6^, ^7^ The paper by Fuller and colleagues^6^ was carried out on a combined match/training group of Hong Kong and English rugby players; this study indicated no significant difference in incidence or severity of injuries between artificial and natural surfaces. Similarly, the study by Ranson et al.^5^ also indicated no greater risk or severity of injuries, more a change in injury patterns between surfaces; this was carried out on two rugby teams across three seasons. Williams et al.^8^ carried out a meta‐analysis of football codes (including rugby union, soccer, and american football) and indicated comparable injury incidence between artificial and natural surfaces. The final key paper discussed here was by Cousins et al.^7^ which described a single team across two leagues (due to promotion/relegation) and indicated increased severity of injuries on artificial surfaces in comparison with natural. While these studies indicated varied findings, they included small samples and/or few playing seasons and thus lacked statistical power, especially when considering specific injury diagnoses and mechanisms. Accordingly, the current study aims to describe the epidemiology of match injuries in professional male rugby players in England on different playing surfaces over a 6‐season period (2013/14–2018/19 inclusive). Specifically, injury incidence, severity, and burden will be compared between artificial and natural/hybrid (natural grass and hybrid combined into one category) surfaces, with a further sub‐group analysis of domestic competition only data comparing artificial, hybrid, and natural grass surfaces as three separate categories.

## 2 MATERIALS AND METHODS

### 2.1 Study design and setting

Participants were recruited as part of the ongoing Professional Rugby Injury Surveillance Project (PRISP). Clubs involved in this project were required to collect injury data as part of the competition minimum requirements. Match exposure data for this analysis were gathered from the 12 Premiership teams each season from the 2013/14 season, when the first artificial grass playing surface was introduced in elite level English men's rugby, up to and including the 2018/19 season. Across these six seasons, a total of 1053.5 eligible matches (artificial 179.5, hybrid 453, natural 421) were used to calculate exposure with 15 teams providing data due to promotion and relegation of clubs during this period.

All consenting players who were members of their club's first team squad were eligible for inclusion. Players were individually consented at the start of every season regardless of whether they had consented in previous seasons. This secondary analysis was approved by the University of Bath's Research Ethics Approval Committee for Health (REACH) (EP 19.20038). Data collection for PRISP also has REACH approval (EP 16.17200). This study's methods and reporting are consistent with the Strengthening the Reporting of Observational Studies in Epidemiology ‐ Sports Injury and Illness Surveillance (STROBE‐SIIS).^9^ Initial venue surface information was extracted from entries made by club medical practitioners in the medical note keeping and injury surveillance platform for the league (Rugby Squad medical, RSM, The Sports Office, UK). A randomly selected 10% of the domestic league injury records were reviewed to compare practitioners' entry accuracy for match venues with confirmed fixture lists.^10^, ^11^, ^12^ One author (CR) compared inputted information with fixture lists as well as publicly available information regarding the playing surface present at the venue. This process indicated that venue entry accuracy was high (94%) on UK match venues and playing surface. For this study, hybrid and natural grass surfaces were combined into one category for the European/domestic data set. This was due to the likelihood of practitioners being easily able to ascertain if a match was played on an artificial surface, but less able to distinguish between hybrid or natural grass surfaces, particularly at less familiar venues.

Analysis of combined domestic league (Premiership) and European match play data was carried out to compare injuries on artificial surfaces with injuries on natural/hybrid surfaces. Natural/hybrid was combined into one category as reliable information on whether surfaces are natural or hybrid for some teams outside the English Premiership was not always available. Only data from English teams competing in European cup competition were included in this analysis. Further analysis was carried out in a subset of domestic league (English Premiership) only data, with comparison between three categories of surface (artificial, hybrid, and natural). This was deemed appropriate due to the more assured knowledge of playing surface for these venues.

### 2.2 Variables/definitions

#### 2.2.1 Injury definition

For the purposes of this study, injury was defined as “an injury which results in at least 24 h of time loss from midnight on the night that the injury occurs,” and definitions align with the international rugby consensus statement for epidemiological studies.^13^ Injuries reported via Rugby Squad Medical (The Sports Office, United Kingdom) were included for analysis if they occurred in first team match play and met the 24‐h time‐loss injury definition.^9^ The Orchard Sports Injury Classification System (OSICS) was used by club medical staff to record injury diagnoses.^14^ Exposure for English Premiership matches and English teams playing in matches in European competition was calculated using official competition fixture lists. Exposure per surface was calculated by establishing a count of the number of matches played and multiplying this by the number of exposed players and then by 1.33 (80‐min game converted to hours).

#### 2.2.2 Statistical methods/data analysis

Injury incidence rate was calculated as the number of injuries per 1000 player‐match‐hours. Mean severity was calculated as the total sum of days absence divided by the total number of injuries, while median severity was calculated as the midpoint of the range of injury severities within data being analyzed. Both the mean and median severity and burden were calculated to account for the potential skew in mean severity caused by a small number of long‐term injuries. Injury burden was calculated as the product of mean severity and incidence rate to give the number of days absence per 1000 player‐match hours. Burden was also calculated as the product of median severity and incidence rate and served as a sensitivity check of significant differences in mean injury burden; that is, whether such differences had been skewed by a small number of severe injury cases. Burden values that were significantly different using both mean and median severity values were considered significant. All estimates are presented with 95% Poisson confidence intervals. Significant differences were assumed if the 95% confidence intervals (CI) for the variables did not overlap and further confirmed with the use of Bonferroni‐corrected two‐tailed z‐tests. Analyses of the data in this manuscript were carried out in Microsoft Windows Excel for Windows 10 and R (version 4.0.5, R Foundation for Statistical Computing).

## 3 RESULTS

### 3.1 Combined domestic/European data: Overall incidence, severity, and burden (Figure 1)

**FIGURE 1:**
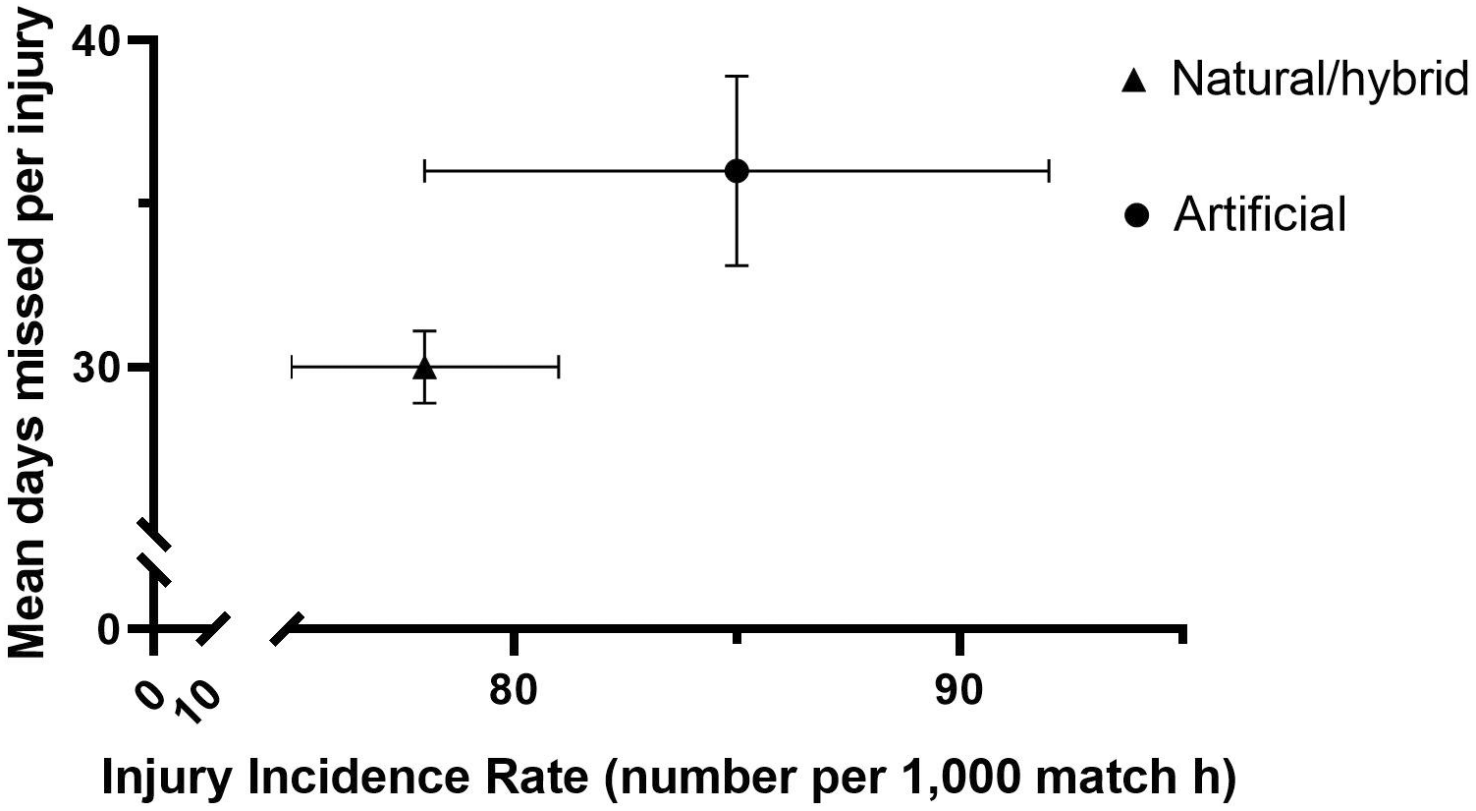
Incidence and mean severity of all injuries across artificial and natural/hybrid surfaces; data for combined European and domestic matches. Data contained in Table S1.

In the combined domestic league and European competition data, there were a total of 3351 injuries (artificial 608; natural/hybrid 2743) in 42 140 h of exposure (artificial 7180 h; natural/hybrid 34 960 h) across six seasons. Incidence rates were not significantly different on each surface (artificial 85/1000 h, 95% CI: 78–92 vs. natural/hybrid 78/1000 h, 95% CI: 76–81, *p* = 0.09) (Figure 1). The mean severity of injuries on artificial surfaces was significantly greater than those on natural/hybrid surfaces (artificial 36 days, 95% CI: 34–39, median 13 days, vs. natural/hybrid 30 days, 95% CI: 29–31, *p* < 0.05, median 11 days). Injury burden was significantly greater on artificial surfaces than natural/hybrid (artificial 3082 days/1000 h, 95% CI: 2847–3337 vs. natural/hybrid 2364 days/1000 h, 95% CI: 2277 – 2454, *p* < 0.001).

Table 1 displays injury incidence per severity category, indicating that the incidence rate for injuries lasting 29–84 day (artificial 18.9/1000 h vs. natural/hybrid 13.2/1000 h) was the only significant difference between surface types here.

**TABLE 1:** Incidence of injuries in different severity groupings, Domestic/European combined data

Surface	Incidence/1000 h (95% CI)				
	2–7 days	8–28 days	29–84 days	>84 days	All injuries
Artificial	26.9 (23.3–31.0)	29.2 (25.6–33.5)	**18.9 (16.0–22.4)** ^a^	8.4 (6.5–10.8)	84.7 (78.2–91.69)
Natural/Hybrid	29.1 (27.4–31.0)	26.9 (25.3–28.7)	13.2 (12.0–14.4)	7.6 (6.8–8.6)	78.5 (75.6–81.5)

^a^ Significantly higher than 29–84 days injuries on natural/hybrid surfaces.

### 3.2 Domestic only data: Overall incidence, severity, and burden (Figure 2)

For the domestic league‐only data, 2675 injuries were recorded during the 6 seasons (artificial 506; hybrid 1224; natural 945) in 32 400 h of exposure (artificial 5800 h; hybrid 15 560 h; natural 11 040 h). No significant differences in injury incidence rates between surfaces were observed (artificial 87/1000 h, 95% CI: 80–95 vs. hybrid 79/1000 h, 95% CI: 74–83 vs. natural 86/1000 h, 95% CI: 80–91). The mean severity of injuries on artificial surfaces (artificial 35 days, 95% CI: 32–38, median 12 days) was significantly greater than on natural surfaces (28 days, 95% CI: 26–30, median 10 days, *p* < 0.001) but not hybrid surfaces (31 days, 95% CI: 29–33, median 11 days, *p* = 0.08). Injury burden was significantly greater on artificial (3041 days/1000 h, 95% CI: 2788 – 3318, *p* < 0.001) compared with both hybrid (2428 days/1000 h, 95% CI: 2295–2567, *p* < 0.001) and natural surfaces (2401 days/1000 h, 95% CI: 2253–2559, *p* < 0.001). Severity category data for the domestic only data set is in Table S4.

**FIGURE 2:**
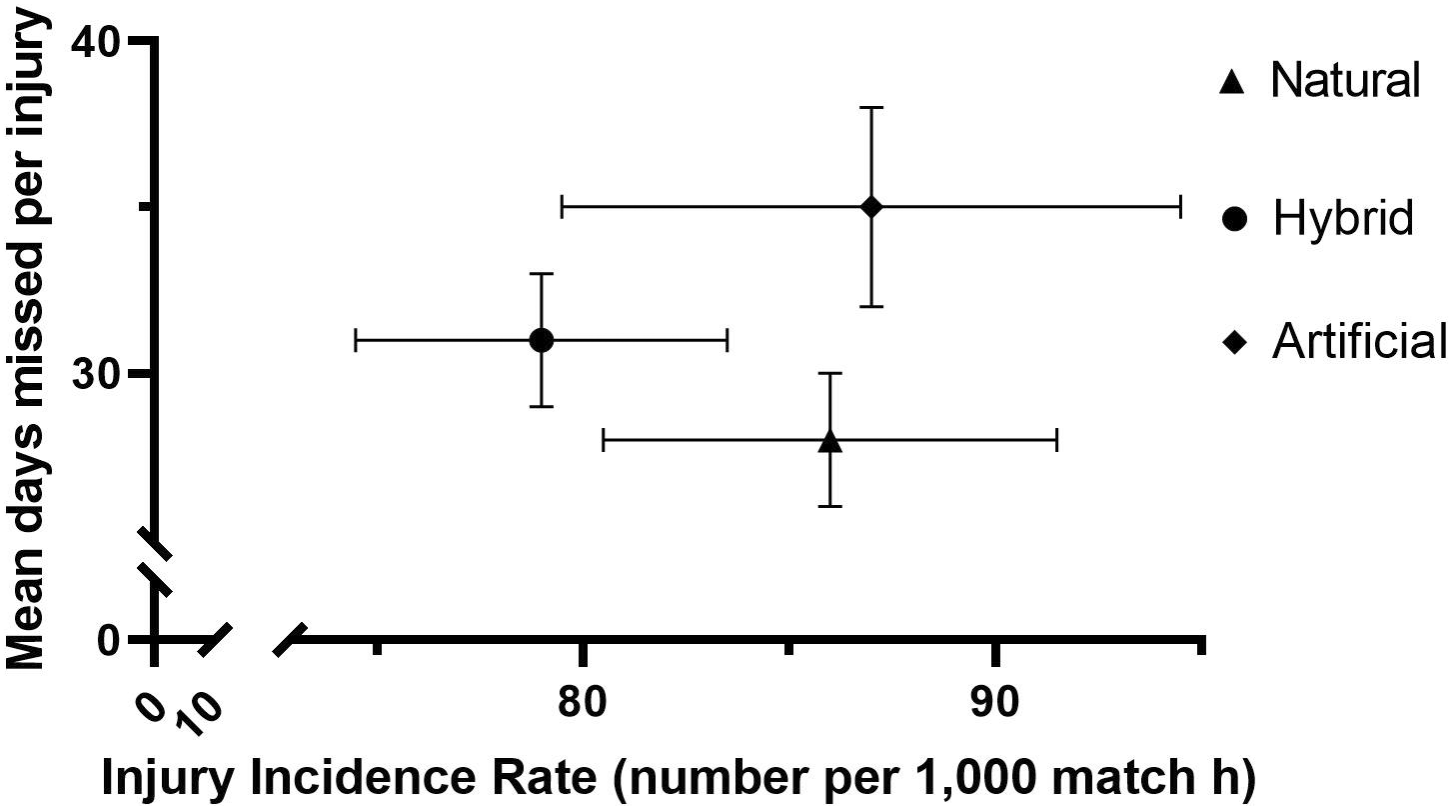
Incidence and mean severity of all injuries sustained in a subgroup of domestic matches from 2013/14 to 2018/19 inclusive

### 3.3 Combined domestic/European data: Injury location (Table 2) and type (Table S2)

When analyzing the combined domestic/European dataset, incidence rates for each body location were similar on different surfaces except for lower leg/achilles tendon, which had a significantly greater incidence on natural/hybrid surfaces (artificial 2.8 /1000 h, 95% CI: 1.8–4.3 vs. Natural/hybrid 5.1 /1000 h, 95%CI: 4.4–5.9, *p* = 0.01) (Table 2). Injuries on artificial surfaces resulted in significantly greater severity for hip/groin (artificial 35 days, 95% CI: 24–51, median 17 days vs. natural/hybrid 18 days, 95% CI: 15–22, median 6 days, *p* = 0.002), foot/toe (artificial 83 days, 95% CI: 56–123, median 38 days vs. natural/hybrid 29 days, 95% CI: 24–36, median 11 days, *p* < 0.001), and neck/cervical spine (artificial 45 days, 95% CI: 29–69, median 8 days vs. natural/hybrid 18 days, 95% CI: 16–22, median 6 days, *p* = 0.04). Mean burden was significantly greater on artificial surfaces for posterior thigh (artificial 252 days/1000 h, 95% CI: 189–335 vs. natural/hybrid 156 days/1000 h, 95% CI: 133–182, *p* < 0.001), hip/groin (artificial 128 days/1000 h, 95% CI: 87–189 vs. natural/hybrid 51 days/1000 h, 95% CI: 42–62, *p* < 0.001), and foot/toe (artificial 290 days/1000 h, 95% CI: 196–429 vs. natural/hybrid 78 days/1000 h, 95% CI: 64–96, *p* < 0.001) injuries. Lower leg/achilles tendon injuries had a greater burden on natural/hybrid (artificial 58 days/1000 h, 95% CI 38–90 vs. natural/hybrid 147 days/1000 h, 95% CI 127–171, *p* < 0.001). There was no significant difference between artificial surface concussion incidence (15.2/1000 h, 95% CI: 12.6–18.3) and natural/hybrid surfaces (14.2/1000 h, 95% CI: 13.1–15.6). Details on all injury type data are contained in Table S2. A key injury of interest when considering surface type is injury to the anterior cruciate ligament (ACL); in this study, there were no significant differences found in incidence (artificial, 0.4/1000 h, 95% CI: 0.1–1.3 vs. natural/hybrid, 0.6/1000 h, 95% CI: 0.4–0.9), severity (artificial, 178 days/1000 h, 95% CI: 57–552 vs. natural/hybrid, 225 days/1000 h, 95% CI: 145–348) or burden (artificial, 74 days/1000 h, 95% CI: 24–231 vs. natural/hybrid, 129 days/1000 h, 95% CI: 83–199) across surfaces for this injury.

**TABLE 2:** Incidence, mean/median severity and mean burden values for injury locations across the 2013/14–2018/19 seasons in domestic and European competition combined data set

Location	Incidence (/1000 h)				Mean Severity (Days)		Mean Burden (Days/1000 h)		Median Severity (Days)	
	N	Artificial (95% CI)	N	Natural/hybrid (95% CI)	Artificial (95% CI)	Natural/hybrid (95% CI)	Artificial (95% CI)	Natural/hybrid (95% CI)	Artificial (IQR)	Natural/hybrid (IQR)
Head/Face	139	19.4 (16.4–22.9)	602	17.2 (15.9–18.7)	15 (13–18)	18 (17–20)	299 (254–353)	316 (292–343)	9 (16–18)	9 (5–15)
Knee	86	12.0 (9.7–14.8)	332	9.5 (8.5–10.6)	59 (48–73)	55 (50–61)	709 (574–876)	524 (470–583)	26 (6–66)	25 (6–69)
Ankle	60	8.4 (6.5–10.8)	268	7.7 (6.8–8.6)	44 (34–57)	44 (39–50)	369 (287–478)	340 (302–383)	17 (5–63)	20 (6–57)
Shoulder/Clavicle	60	8.4 (6.5–10.8)	299	8.6 (7.6–9.6)	44 (35–57)	39 (35–44)	371 (288–478)	335 (299–375)	19 (5–54)	14 (45–46)
Posterior thigh	47	6.6 (4.9–8.7)	156	4.5 (3.8–5.2)	38 (29–51)	35 (30–41)	**251 (189–335)** ^a^	155 (133–182)	27 (9–59)	22 (11–42)
Hand/Finger/Thumb	32	4.5 (3.2–6.3)	115	3.3 (2.7–4.0)	28 (20–40)	31 (26–38)	125 (89–177)	103 (86–124)	23 (5–43)	22 (5–43)
Hip/groin	26	3.6 (2.5–5.3)	99	2.8 (2.3–3.5)	**35 (24–52)** ^a^	18 (15–22)	**128 (87–189)** ^a^	51 (42–62)	17 (8–33)	6 (5–17)
Sternum/Ribs/Upper Back	26	3.6 (2.5–5.3)	127	3.6 (3.1–4.3)	16 (11–23)	14 (11–16)	58 (39–85)	49 (41–58)	13 (7–23)	9 (4–20)
Foot/toe	25	3.5 (2.4–5.2)	94	2.7 (2.2–3.3)	**83 (56–123)** ^a^	29 (24–36)	**290 (196–429)** ^a^	78 (64–96)	38 (6–115)	11 (4–29)
Anterior Thigh	25	3.5 (2.4–5.2)	136	3.9 (3.3–4.6)	14 (9–20)	10 (8–12)	48 (32–71)	38 (32–45)	9 (4–18)	5 (3–10)
Neck/Cervical Spine	21	2.9 (1.9–4.5)	143	4.1 (3.5–4.8)	**45 (30–69)** ^a^	18 (16–22)	131 (86–201)	75 (64–88)	8 (4–16)	6 (4–16)
Lower leg/Achilles tendon	20	2.8 (1.8–4.3)	178	**5.1 (4.4–5.9)** ^a^	21 (13–32)	29 (25–34)	58 (38–90)	**147 (127–171)** ^a^	10 (7–39)	11 (4–23)
Elbow	12	1.7 (1.0–2.9)	50	1.4 (1.1–1.9)	22 (13–39)	27 (21–36)	37 (21–66)	39 (29–51)	5 (4–52)	14 (5–35)
Low Back	11	1.5 (0.9–2.8)	63	1.8 (1.4–2.3)	12 (6–21)	15 (12–19)	18 (10–32)	27 (21–35)	6 (3–22)	8 (4–16)
Upper Arm	9	1.3 (0.7–2.4)	33	0.9 (0.7–1.3)	114 (59–219)	62 (44–87)	143 (74–274)	58 (41–82)	82 (6–199)	54 (5–97)
Abdomen	5	0.7 (0.3–1.7)	27	0.8 (0.5–1.1)	8 (3–20)	13 (9–19)	6 (2–14)	10 (7–15)	8 (4–13)	8 (5–21)
Thigh	3	0.4 (0.1–1.3)	6	0.2 (0.1–0.4)	60 (19–186)	21 (9–47)	25 (8–78)	4 (2–8)	11 (8–161)	24 (7–33)
Pelvis/Sacrum	1	0.1 (0.0–1.0)	12	0.3 (0.2–0.6)	106 (15–753)	31 (18–54)	15 (2–105)	11 (6–19)	106 (n/a)	14 (6–48)

^a^ Significantly higher than its associated value on the opposite surface. Burden values are only noted as significant if they remain so following the median severity value sensitivity check.

### 3.4 Domestic only data: Injury location (Table S5) and type (Table S6)

Injury burden on artificial surfaces was also greater for some specific locations when separated in to three surfaces in the sub‐analysis. For posterior thigh, injury burden on artificial surfaces (279 days/1000 h, 95% CI: 205–380) was significantly greater than natural (147 days/1000 h, 95% CI: 110–194, *p* < 0.001), and hybrid surfaces (162 days/1000 h, 95% CI: 129–205, *p* = 0.03). For hip/groin, injury burden on artificial surfaces (156 days/1000 h, 95% CI: 106–231) was significantly greater than on both hybrid (50 days/1000 h, 95% CI: 38–66, *p* < 0.001) and natural grass surfaces (54 days/1000 h, 95% CI: 37–78, *p* < 0.001). For foot/toe, injury burden on artificial surfaces (315 days/1000 h, 95% CI: 208–479) was significantly greater than on both hybrid (123 days/1000 h, 95% CI: 94–162, *p* < 0.001) and natural grass surfaces (43 days/1000 h, 95% CI: 28–65, *p* < 0.001). For upper arm, injury burden was greater on artificial surfaces (163 days/1000 h, 95% CI: 81–325) than on hybrid (28 days/1000 h, 95% CI: 17–45, *p* < 0.001) but not natural grass surfaces (76 days/1000 h, 95% CI: 41–140, *p* = 0.32).

Further to the above, injury type information also indicated that burden of some injuries was significantly different within the three‐surface analysis. Muscle strain/rupture/cramps on artificial surfaces were significantly greater than those on both hybrid and natural playing surface surfaces (artificial, 438 days/1000 h, 95% CI: 353–544 vs. hybrid, 304 days/1000 h, 95% CI: 263–351 vs. natural, 211 days/1000 h, 95% CI: 177–251). Dislocation/subluxation injuries had significantly greater burden on artificial and natural grass surfaces in comparison to hybrid surfaces, but not when compared with each other (artificial, 278 days/1000 h, 95% CI: 165–469 vs. hybrid, 93 days/1000 h, 95% CI: 65–132 vs. natural, 223 days/1000 h, 95% CI: 151–327).

### 3.5 Combined domestic/European data: Match event (Table 3)

There were no differences between surfaces in injury incidence rates, severity, or burden across different known match events however there were significant differences noted in both the “unknown” and “other” categories. Similar to those injuries in the domestic only data set, no pattern of characteristics was noted within “other” or “unknown” here. “Other” refers to injuries which the medical staff entering the data felt did not fit in available game event categories. “Unknown” refers to those injuries which the medical staff could not ascertain the specific event causing the injury. This is not uncommon in sports such as rugby due to the intensity of match play, multifactorial nature of many injuries and variable visibility available to medical staff when observing match play live or on video following the event.

**TABLE 3:** Incidence, mean/median severity, and mean burden values associated with match events recorded as the activity at point of onset of a time loss injury

Event	Incidence (/1000 h)				Mean Severity (Days)		Mean Burden (Days/1000 h)		Median Severity (Days)	
	N	Artificial (95% CI)	N	Natural/Hybrid (95% CI)	Artificial (95% CI)	Natural/Hybrid (95% CI)	Artificial (95% CI)	Natural/Hybrid (95% CI)	Artificial (IQR)	Natural/Hybrid (IQR)
Tackled	147	20.5 (17.4–24.1)	663	19.0 (17.6–20.5)	29 (25–34)	33 (31–36)	595 (507–700)	630 (583–679)	12 (5–35)	11 (5–34)
Tackling	138	19.2 (16.3–22.7)	579	16.6 (15.3–18.0)	42 (35–41)	30 (28–33)	800 (677–945)	499 (460–541)	13 (6–38)	11 (5–30)
Collisions	64	8.9 (7.0–11.4)	352	10.1 (9.1–11.2)	28 (22–35)	23 (21–25)	246 (192–314)	230 (207–256)	12 (6–29)	9 (5–24)
Running	55	7.7 (5.9–10.0)	255	7.3 (6.5–8.2)	50 (39–65)	47 (42–53)	385 (295–501)	343 (303–388)	22 (10–54)	17 (8–40)
Ruck	41	5.7 (4.2–7.8)	259	7.4 (6.6–8.4)	38 (28–51)	33 (29–37)	215 (158–292)	243 (215–275)	16 (6–50)	11 (5–41)
Scrum^b^	14	3.7 (2.2–6.2)	75	4.0 (3.2–5.0)	67 (40–113)	37 (30–47)	245 (145–414)	149 (119–187)	31 (4–89)	11 (5–36)
Lineout	9	1.3 (0.7–2.4)	34	1.0 (0.7–1.4)	26 (14–50)	28 (20–39)	33 (17–63)	27 (19–38)	29 (11–33)	24 (6–47)
Maul	8	1.1 (0.6–2.2)	65	1.9 (1.5–2.4)	20 (10–39)	20 (16–26)	22 (11–44)	39 (30–48)	16 (4–21)	11 (5–24)
Kicking	3	0.4 (0.1–1.3)	9	0.2 (0.1–0.5)	50 (16–156)	47 (24–90)	21 (7–65)	12 (6–23)	27 (3–121)	23 (5–31)
Other	40	**5.6 (4.1–7.6)** ^a^	113	3.2 (2.7–3.9)	**38 (28–52)** ^a^	16 (13–19)	**212 (156–289)** ^a^	52 (43–62)	14 (5–44)	6 (4–15)
Unknown	87	12.1 (9.8–15.0)	330	9.4 (8.5–10.5)	**34 (28–42)** ^a^	22 (20–24)	**417 (338–515)** ^a^	205 (184–229)	9 (4–34)	9 (4–22)

^a^ Significantly higher than its associated value on the opposite surface. Burden values are ONLY noted as significant here if they remain so following the median severity value sensitivity check.

^b^ Scrum exposure has been modified to reflect this set piece only impacting forward (8/15 × full exposure per surface).

### 3.6 Domestic only data: Match event (Table S7)

Calculations using mean severity values indicated greater burden from injuries sustained in tackling on artificial surfaces (777 days/1000 h, 95% CI 650–928) compared with both other surface types (hybrid 505 days/1000 h, 95% CI 446–572, *p* < 0.001 vs. natural 546 days/1000 h, 95% CI 476–627, *p* = 0.007).

## 4 DISCUSSION

### 4.1 Key findings

The aim of this study was to undertake the largest investigation to date of the influence of playing surface type on match injuries in professional men's rugby union. Overall, injury incidence rates in this study were not significantly different between the different playing surfaces. However, there were differences in mean severity and mean burden of injuries sustained between playing surfaces (Figures 1 and 2). These findings were consistent when comparing artificial with combined natural/hybrid playing surfaces (European/domestic data) and when comparing artificial with hybrid and natural grass surfaces separately (domestic only data).

### 4.2 Incidence of injury

The overall incidence of injury was not significantly different when comparing surface types in this study (artificial 85/1000 h, 95% CI: 78–92 vs. natural/hybrid 78/1000 h, 95% CI: 76–81, *p* = 0.09). This finding is supported by research conducted in another professional cohort from the UK (artificial, 80.2/1000 h, 95% CI: 69.9–91.7 vs. grass 81.9/1000 h, 95% CI: 72.2–92.5)^5^ and an amateur cohort in Hong Kong (artificial 38.2/1000 h, 95% CI: 29.1–50.1 vs. grass 26.9/1000 h, 95% CI: 18.6–39.0)^6^ as well as systematic reviews of surface types.^8^, ^15^ Conversely, one more recent study reported substantially different incidence rates for natural grass (82.8/1000 h) and for artificial surfaces (160.3/1000 h).^7^ This discrepancy highlights inconsistencies surrounding injury incidence between surfaces when grouped into two categories of artificial and natural/hybrid. Critical review of the above studies highlights the lack of statistical power with limited participant numbers. The current study draws from a larger pool of participant data and so can produce narrower confidence intervals and more assured conclusions regarding the influence of surface type on injury.

### 4.3 Severity of injury

Previous studies in rugby have not reported significant differences in the mean severity of injuries on different surface types.^5^, ^6^, ^7^, ^8^ Conversely, this analysis found significant increases in mean severity of injuries sustained on artificial surfaces in comparison to natural/hybrid surfaces in a European/domestic dataset as well as domestic only matches. This could be explained by a greater number of 29–84 day injuries (Table 1) as well as the greater severity of some specific injury locations; posterior thigh, hip/groin, and foot/toe (Table 2). Interestingly, when natural, hybrid, and artificial surfaces were compared as three categories, there was no significant difference between hybrid surfaces and either natural grass or artificial turf. The significant difference in the domestic only dataset came between artificial and natural grass with artificial surfaces producing the more severe injuries.

### 4.4 Injury patterns

When considering injury location on different surfaces, this study suggests an overall greater mean severity and burden in lower limb injuries on artificial surfaces. Of these, specific locations of the hip/groin, posterior thigh and foot/toe were highlighted as the primary sites of this increase on artificial surfaces when compared with other surface types. The reason for this increase has been hypothesized; greater torque and rotational stiffness experienced in lower limbs on such surfaces have been suggested as the rationale for this,^16^ particularly in certain surface/boot combinations with injury location and mechanism being discussed in multiple studies on National Football League (NFL)^17^, ^18^, ^19^ and elite level Soccer cohorts.^20^, ^21^ A previous analysis in the NFL from 2000 to 2009 indicated greater risk in both knee and ankle sprains on artificial surfaces with proper maintenance and development of surface technology being noted as important factors to consider in ensuring optimal pitch condition was maintained to mitigate some preventable injuries.^19^ While artificial pitch technology has undoubtedly progressed since this period, it highlights the importance of ongoing review of surface types in sport. More recently, the NFL has directed significant investigation into the influence of boot‐surface interaction and on forces applied to athletes during match play.^17^ This relationship may have a key role to play in terms of dissipation of force along the lower limb kinetic chain and therefore could impact on the severity of injuries sustained. Without adequate force dissipation, greater rotational and shear forces may be applied to lower limb tissues. This greater force may exceed the capacity of those structures to a greater extent than on grass and therefore modify the subsequent severity of injuries sustained on artificial surfaces,^17^, ^18^ although it should be acknowledged that there is wide variation in the properties of natural surfaces. A recent, mechanistic analysis^22^ of boot‐surface interaction may offer some further support of this theory. The review suggested that on contact between the players' footwear and artificial pitch surface, the pitch infill compresses and becomes denser. As a result, the player will experience an increase in peak forces and different loading rates compared with a natural grass surface.^22^ Given the above discussed findings as well as the overall findings of the current study suggesting a greater mean severity and burden of lower limb injuries on artificial surfaces, investigation into the interaction of athlete's feet, footwear, and surface in rugby is warranted as has been suggested.^5^, ^7^

Certain specific diagnoses have been highlighted as being of interest in previous studies. For example, anterior cruciate ligament (ACL) injuries have been reported to have a fourfold increase in incidence on artificial playing surfaces in comparison to natural grass. This was based on 5 injuries within the dataset, and it was noted by Fuller^6^ that these results were of no statistical significance due to the low numbers of this injury within the study. Despite this acknowledgment, the ACL is a high time loss injury that impacts many sports, and we believe is worth discussing specifically here. The findings in this study do not indicate any significant difference in incidence, mean severity, or burden of match play ACL injuries between playing surfaces. Similarly, here, the sample size was small with a total of three ACL injuries on artificial surfaces and 20 injuries on natural/hybrid surfaces reported over the six seasons of this study; While this study contains a large amount of data, further detailed analysis (e.g., specific injury diagnoses) is still limited by small numbers of the less common injuries available at the current time. Bahr and Holme noted in 2003 that 20–50 injury cases are required to detect moderate‐to‐strong associations between data groups and 200 injury cases required to detect small to moderate associations^23^ meaning it is challenging to carry out meaningful sub‐group analysis with the volume of data available at the present time.

Concussion has also been reported as having significantly greater incidence rates when matches take place on natural grass surfaces when compared to artificial surfaces^5^; however, the present study did not identify any differences in incidence, mean severity, or burden of concussion across playing surfaces.

### 4.5 Limitations

It is noted within the IOC epidemiological reporting consensus^9^ and previous gold standard longitudinal studies of this size^3^ that there are potential challenges associated with collecting multi‐team, longitudinal epidemiological data. For instance, changes to the research team, data input methods, and multiple‐party involvement in terms of system management, can all lead to variance in the consistency and resultant quality of a data set. To address these issues, the current study used a number of quality control processes. Data input was undertaken by qualified clinical staff using a bespoke electronic medical notes system (RSM), and final approval of the included injuries was obtained from the medical lead in each club. Furthermore, all data exported were checked for duplicates and inconsistencies, and the playing surface type at each venue was confirmed. These practices help to maintain data quality and are in‐line with the IOC reporting consensus.^9^ Additionally, it is noted that factors such as team playing style, playing surface maintenance behaviors, and playing surface quality will influence the injury risk at a given venue regardless of playing surface type, but that these factors were not fully accounted for in the analysis. Finally, given the number of comparisons made, the overall Type I error rate was potentially inflated beyond the 5% level. This effect was attenuated by the use of Bonferroni corrections, as well as the use of median severity values as a sensitivity analysis for any clear differences in injury burden between playing surfaces.

## 5 PERSPECTIVE

This study of six seasons of domestic and European data is the largest of its kind, examining epidemiological data of injuries across playing surfaces commonly used for matches in rugby union. While injury rate between surfaces was not significantly different, overall injury severity was significantly greater when an injury was sustained on artificial surfaces in comparison with either combined natural/hybrid surface type or when natural grass was separated from hybrid. Of the above noted outcomes, significant findings were predominantly lower limb issues and regions that may be impacted by ground reaction force relationships and traction. The installation of artificial pitches undoubtedly allows for greater repeated use of the surface as well as reduced variability in hardness due to weather changes; however, these benefits must be weighed up against the potential for greater injury burden, particularly in the elite setting examined here. Injury data should be reviewed regularly in order to evaluate changes in injury patterns as pitch technology evolves.

## CONFLICT OF INTEREST

SWi and KS have received funding for this research as part of the Professional Rugby Injury Surveillance Project (PRISP), joint funded by the Rugby Football Union (RFU), and Premiership Rugby. LS is a PhD student funded as part of the PRISP group with her affiliations listed. CR is a PhD student funded jointly by the Rugby Football Union, Premiership Rugby and University of Bath. SK, KS, and MC are employed within rugby union in the United Kingdom with their institutional affiliations listed.

## Supporting information


Tables S1‐S7

## Data Availability

All publicly available data are included in the article or uploaded as online Tables S1–S7.

## References

[1] SIS Pitches . Rugby Pitches. 03/02/2021, 2021. Accessed 03/02/2021. https://www.sispitches.com/ie/your‐sport/rugby‐pitches/

[2] Roberts SP , Trewartha G , Higgitt RJ , El‐Abd J , Stokes KA . The physical demands of elite English rugby union. J Sports Sci. 2008;26(8):825‐833. doi:10.1080/02640410801942122 18569548

[3] West SW , Starling L , Kemp S , et al. Trends in match injury risk in professional male Rugby union: a 16‐season review of 10 851 match injuries in the English premiership (2002–2019): the professional Rugby injury surveillance project. Br J Sports Med. 2021;55(12):676‐682. doi:10.1136/bjsports-2020-102529 33046453

[4] Williams S , Trewartha G , Kemp SP , Michell R , Stokes KA . The influence of an artificial playing surface on injury risk and perceptions of muscle soreness in elite Rugby union. Scand J Med Sci Sports. 2016;26(1):101‐108. doi:10.1111/sms.12402 25644277

[5] Ranson C , George J , Rafferty J , Miles J , Moore I . Playing surface and UK professional rugby union injury risk. J Sports Sci. 2018;36(21):2393‐2398. doi:10.1080/02640414.2018.1458588 29595083

[6] Fuller CW , Clarke L , Molloy MG . Risk of injury associated with rugby union played on artificial turf. J Sports Sci. 2010;28(5):563‐570. doi:10.1080/02640411003629681 20391085

[7] Cousins BEW , Morris JG , Sunderland C , Bennett AM , Shahtahmassebi G , Cooper SB . Synthetic playing surfaces increase the incidence of match injuries in an elite Rugby union team. J Sci Med Sport. 2021;25:134‐138. doi:10.1016/j.jsams.2021.08.019 34538756

[8] Williams S , Hume PA , Kara S . A review of football injuries on third and fourth generation artificial turfs compared with natural turf. Sports Med. 2011;41(11):903‐923. doi:10.2165/11593190-000000000-00000 21985213

[9] Bahr R , Clarsen B , Derman W , et al. International Olympic Committee consensus statement: methods for recording and reporting of epidemiological data on injury and illness in sport 2020 (including STROBE extension for sport injury and illness surveillance (STROBE‐SIIS)). Br J Sports Med. 2020;54(7):372‐389. doi:10.1136/bjsports-2019-101969 32071062 PMC7146946

[10] Premiership Rugby . Gallagher Premiership Rugby Fixtures. 2021. Accessed 03/02/2021, 2021. https://www.premiershiprugby.com/gallagher‐premiership‐rugby/fixtures‐results/

[11] European Cup Rugby . European Champions Cup Rugby Fixtures. 2021, Accessed 03/02/2021. https://www.epcrugby.com/champions‐cup/matches/fixtures‐and‐results/

[12] European Cup Rugby . European Challenge Cup Rugby Fixtures. 2021. Accessed 03/02/2021, 2021. https://www.epcrugby.com/challenge‐cup/matches/fixtures‐and‐results/

[13] Fuller CW , Molloy MG , Bagate C , et al. Consensus statement on injury definitions and data collection procedures for studies of injuries in rugby union. Br J Sports Med. 2007;41(5):328‐331. doi:10.1136/bjsm.2006.033282 17452684 PMC2659070

[14] Orchard JW , Meeuwisse W , Derman W , et al. Sport medicine diagnostic coding system (SMDCS) and the Orchard sports injury and illness classification system (OSIICS): revised 2020 consensus versions. Br J Sports Med. 2020;54(7):397‐401. doi:10.1136/bjsports-2019-101921 32114487

[15] Sivasundaram L , Mengers S , Paliobeis A , et al. Injury risk among athletes on artificial turf: a review of current literature. Curr Orthop Pract. 2021;32(5):512‐517. doi:10.1097/BCO.0000000000001021

[16] Taylor SA , Fabricant PD , Khair MM , Haleem AM , Drakos MC . A review of synthetic playing surfaces, the shoe‐surface Interface, and lower extremity injuries in athletes. Phys Sportsmed. 2012;40(4):66‐72. doi:10.3810/psm.2012.11.1989 23306416

[17] Jastifer JR , McNitt AS , Mack CD , et al. Synthetic turf: history, design, maintenance, and athlete safety. Sports Health. 2019;11(1):84‐90. doi:10.1177/1941738118793378 30096021 PMC6299344

[18] Jastifer J , Kent R , Crandall J , et al. The athletic shoe in football. Sports Health. 2017;9(2):126‐131. doi:10.1177/1941738117690717 28151702 PMC5349396

[19] Hershman EB , Anderson R , Bergfeld JA , et al. An analysis of specific lower extremity injury rates on grass and FieldTurf playing surfaces in National Football League Games. Am J Sports Med. 2012;40(10):2200‐2205. doi:10.1177/0363546512458888 22972855

[20] Thomson A , Whiteley R , Bleakley C . Higher shoe‐surface interaction is associated with doubling of lower extremity injury risk in football codes: a systematic review and meta‐analysis. Br J Sports Med. 2015;49(19):1245‐1252. doi:10.1136/bjsports-2014-094478 26036677

[21] Thomson A , Rennie D . Evolution of natural grass playing surfaces for elite football. Aspetar Sport Med J. 2016;5:322‐327.

[22] Forrester S , Fleming P . Traction forces generated during studded boot‐surface interactions on third‐generation artificial turf: a novel mechanistic perspective. Eng Rep. 2019;1(5):e12066. doi:10.1002/eng2.12066

[23] Bahr R . Risk factors for sports injuries ‐‐ a methodological approach. Br J Sports Med. 2003;37(5):384‐392. doi:10.1136/bjsm.37.5.384 14514527 PMC1751357

