# Supplementary material for: Influence of playing surface on match injury risk in men's professional rugby union in England (2013–2019)
Source: Scand J Med Sci Sports. 2022 Sep 4;32(11):1615–24. doi: 10.1111/sms.14226 (PMC9825862; doi:10.1111/sms.14226)
Supplement: Supplementary file 1 — Tables S1‐S7 [file SMS-32-1615-s001.docx]

**SUPPLEMENTARY TABLES**

**Supplementary table 1 – Domestic and European combined overall values**

| **All Injuries** | **Number** | | **Exposure (hrs)** | **Incidence (/1000 hrs)** | **Total time lost (Days)** | **Mean severity** | **Burden (days /1000hrs)** | **Median Severity** |
| --- | --- | --- | --- | --- | --- | --- | --- | --- |
|  | **Count** | **%** |  |  |  |  |  |  |
| **Artificial** | 608 | 18 | 7180 | 85 (78.2 - 92.0) | 19997 | ***33 (30-36)** | ***2785 (2572-3016)** | 13 (32) |
| **Natural/Hybrid** | 2743 | 82 | 34960 | 78 (75.6 - 81.6) | 75897 | 28 (27-29) | 2171 (2091-2254) | 11 (25) |
| **Overall** | 3351 | 100 | 42140 | 80 (76.9 - 82.3) | 95894 | 29 (28-30) | 2276 (2200-2354) | 11 (26) |

**Supplementary Table 2 – Domestic and European combined injury ‘Type’ values**

| **Type** | **Incidence** | | | | **Mean Severity** | | **Burden** | | **Median Severity** | |
| --- | --- | --- | --- | --- | --- | --- | --- | --- | --- | --- |
|  | **N** | **Artificial** | **N** | **Natural/Hybrid** | **Artificial** | **Natural/Hybrid** | **Artificial** | **Natural/Hybrid** | **Artificial** | **Natural/Hybrid** |
| **Sprain/ligament injury** | 160 | 22.3 (19.1 - 26.0) | 706 | 20.2 (18.8 - 21.7) | 38 (32 - 44) | 38 (35 - 41) | 836 (716 - 977) | 761 (707 - 819) | 19 (58) | 16 (45) |
| **Concussion** | 109 | 15.2 (12.6 - 18.3) | 498 | 14.2 (13.1 - 15.6) | 13 (11 - 16) | 16 (14 - 17) | 203 (168 - 245) | 224 (205 - 245) | 10 (11) | 9 (8) |
| **Muscle Strain/rupture/cramps** | 96 | 13.0 (11 - 16) | 398 | 11.0 (10 - 13) | 31 (26 - 38) | 24 (22 - 27) | ***416 (341 - 508)** | 276 (251 - 305) | 19 (33) | 15 (26) |
| **Haematoma/contusion** | 78 | 10.9 (8.7 - 13.6) | 390 | 11.2 (10.1 - 12.3) | ***11 (9 - 14)** | 8 (7 - 8) | 122 (98 - 153) | 84 (76 - 93) | 7 (10) | 5 (6) |
| **Fracture** | 37 | 5.2 (3.7 - 7.1) | 128 | 3.7 (3.1 - 4.4) | 67 (49 - 93) | 57 (48 - 68) | 346 (251 - 477) | 209 (176 - 248) | 43 (65) | 44 (63) |
| **Other** | 34 | 4.7 (3.4 - 6.6) | 155 | 4.4 (3.8 - 5.2) | 29 (21 - 40) | 21 (18 - 24) | 137 (98 - 191) | 92 (78 - 107) | 15 (24) | 10 (22) |
| **Tendon Injury/rupture/tendonosis/bursitis** | 34 | 4.7 (3.4 - 6.6) | 184 | 5.3 (4.6 - 6.1) | 42 (30 - 59) | 33 (28 - 38) | 199 (142 - 278) | 172 (149 - 199) | 12 (33) | 10 (25) |
| **Dislocation/subluxation** | 19 | 2.6 (1.7 - 4.2) | 69 | 2.0 (1.6 - 2.5) | 106 (68 - 166) | 77 (61 - 97) | 281 (179 - 440) | 152 (120 - 192) | 83 (172) | 44 (109) |
| **Lesion to cartilage/meniscus/disk** | 16 | 2.2 (1.4 - 3.6) | 73 | 2.1 (1.7 - 2.6) | 64 (39 - 105) | 58 (46 - 73) | 143 (88 - 234) | 121 (96 - 152) | 32 (117) | 32 (66) |
| **Nerve Injury** | 16 | 2.2 (1.4 - 3.6) | 90 | 2.6 (2.1 - 3.2) | 41 (25 - 66) | 22 (18 - 27) | 90 (55 - 148) | 57 (46 - 70) | 8 (11) | 9 (13) |
| **Laceration/abrasion** | 6 | 0.8 (0.4 - 1.9) | 25 | 0.7 (0.5 - 1.1) | 4 (2 - 10) | 7 (5 - 11) | 4 (2 - 8) | 5 (4 - 8) | 27 (3) | 76 (7) |
| **Other bone injury** | 2 | 0.3 (0.1 - 1.1) | 7 | 0.2 (0.1 - 0.4) | 27 (7 - 108) | 52 (25 - 108) | 8 (2 - 30) | 10 (5 - 22) | 6 (n/a) | 5 (74) |

**Supplementary table 3 – Domestic league overall injury values**

| **All Injuries** | **Number** | | **Exposure (Hrs)** | **Incidence (/1000 Hrs** | **Total time lost (Days)** | **Mean severity** | **Burden (days /1000hrs** | **Median Severity** |
| --- | --- | --- | --- | --- | --- | --- | --- | --- |
|  | Count | % |  |  |  |  |  |  |
| **Artificial** | 505 | 19 | 5800 | 87 (80 - 95) | 17636 | ***35 (32 - 38)** | ***3041 (2787 - 3318)** | 12 (31) |
| **Hybrid** | 1214 | 46 | 15560 | 78 (74 - 83) | 37630 | 31 (29 - 33) | 2418 (2286 - 2558) | 11 (28) |
| **Natural** | 941 | 35 | 11040 | 85 (80 - 91) | 26445 | 28 (26 - 30) | 2395 (2247 - 2553) | 10 (24) |
| **Overall** | 2660 | 100 | 32400 | 82 (79 - 85) | 81711 | 31 (30-32) | 2522 (2428 - 2620) | 11 (26) |

**Supplementary table 4 – Incidence of injuries in different severity groupings, Domestic league data**

|  | Incidence/1000hrs (95% CI) | | | | |
| --- | --- | --- | --- | --- | --- |
| Surface | 2-7 days | 8-28 days | 29-84 days | >84 days | All injuries |
| Artificial | 29.5 (25.4-34.3) | 29.7 (25.5-34.4) | 18.6 (15.4-22.5) | 8.3 (6.4-11.0) | 87.4 (80.1-95.4) |
| Hybrid | 28.0 (25.5-30.7) | 27.4 (24.9-30.1) | 14.1 (12.4-16.1) | 7.7 (6.5-9.2) | 78.6 (74.3-83.1) |
| Natural | 35.4 (32.1-39.1) | 26.7 (23.8-30.0) | 13.3 (11.3-15.7) | 8.1 (6.6-9.9) | 85.6 (80.3-91.2) |

**Supplementary table 5 – Domestic league injury ‘Location’ values**

| **Location** | **Incidence (/1000hrs)** | | | | | | **Mean Severity** | | | | **Burden** | | | **Median Severity** | | |
| --- | --- | --- | --- | --- | --- | --- | --- | --- | --- | --- | --- | --- | --- | --- | --- | --- |
|  | **N** | **Artificial** | **N** | **Hybrid** | **N** | **Natural** | **Artificial** | **Hybrid** | **Natural** | **Artificial** | | **Hybrid** | **Natural** | **Artificial** | **Hybrid** | **Natural** |
| **Head/face** | 120 | 20.7 (17.3 - 24.7) | 267 | 17.2 (15.2 - 19.3) | 211 | 19.1 (16.7 - 21.8) | 15 (12 - 17) | 18 (16 - 20) | 17 (15 - 19) | 302 (252 - 361) | | 303 (269 - 341) | 319 (279 - 365) | 9 | 9 | 9 |
| **Knee** | 66 | 11.4 (8.9 - 14.5) | 156 | 10.0 (8.6 - 11.7) | 104 | 9.4 (7.8 - 11.4) | 40 (31 - 51) | 48 (41 - 56) | 48 (39 - 58) | 453 (356 - 577) | | 477 (408 - 558) | 450 (371 - 545) | 22 | 35 | 13 |
| **Shoulder/clavicle** | 48 | 8.3 (6.2 - 11.0) | 131 | 8.4 (7.1 - 10.0) | 108 | 9.8 (8.1 - 11.8) | 35 (26 - 46) | 27 (23 - 32) | 38 (31 - 46) | 288 (217 - 383) | | 225 (190 - 267) | 371 (307 - 447) | 21 | 15 | 15 |
| **Ankle** | 46 | 7.9 (5.9 - 10.6) | 120 | 7.7 (6.5 - 9.2) | 93 | 8.4 (6.9 - 10.3) | 32 (23 - 42) | 37 (31 - 44) | 37 (31 - 46) | 251 (188 - 335) | | 283 (236 - 338) | 315 (257 - 386) | 12 | 20 | 16 |
| **Posterior Thigh** | 40 | 6.9 (5.1 - 9.4) | 72 | 4.6 (3.7 - 5.8) | 48 | 4.3 (3.3 - 5.8) | 38 (28 - 52) | 34 (27 - 43) | 25 (19 - 33) | ***261 (191 - 355)** | | 157 (125 - 198) | 109 (82 - 145) | 28 | 22 | 17 |
| **hand/finger/thumb** | 28 | 4.8 (3.3 - 7.0) | 47 | 3.0 (2.8 - 4.0) | 42 | 3.8 (2.8 - 5.2) | 24 (16 - 34) | 34 (26 - 46) | 29 (21 - 39) | 114 (78 - 165) | | 104 (78 - 138) | 109 (80 - 147) | 17 | 27 | 15 |
| **Hip/groin** | 25 | 4.3 (2.9 - 6.4) | 48 | 3.1 (2.3 - 4.1) | 28 | 2.5 (1.8 - 3.7) | **36 (24 - 53)** | 16 (12 - 22) | 13 (9 - 18) | ***155 (105 - 229)** | | 50 (37 - 66) | 32 (22 - 46) | 18 | 6 | 6 |
| **Foot/toe** | 22 | 3.8 (2.5 - 5.8) | 52 | 3.3 (2.6 - 4.4) | 22 | 2.0 (1.3 - 3.0) | **70 (46 - 106)** | 29 (22 - 38) | 13 (8 - 19) | ***265 (174 - 402)** | | 97 (74 - 127) | 25 (17 - 38) | 26 | 12 | 8 |
| **Neck/cervical spine** | 20 | 3.4 (2.2 - 5.3) | 53 | 3.4 (2.6 - 4.5) | 59 | 5.3 (4.1 - 6.9) | 23 (15 - 35) | 20 (16 - 27) | 13 (10 - 16) | 78 (51 - 121) | | 69 (53 - 91) | 68 (53 - 88) | 8 | 6 | 5 |
| **Sternum/ribs/upper back** | 19 | 3.3 (2.1 - 5.1) | 54 | 3.5 (2.7 - 4.5) | 30 | 2.7 (1.9 - 3.9) | 15 (10 - 24) | 13 (10 - 16) | 16 (11 - 23) | 50 (32 - 78) | | 44 (34 - 57) | 44 (31 - 63) | 12 | 10 | 8 |
| **Anterior Thigh** | 17 | 2.9 (1.8 - 4.7) | 56 | 3.6 (2.8 - 4.7) | 52 | 4.7 (3.6 - 6.2) | 13 (8 - 20) | 9 (7 - 12) | 8 (6 - 11) | 37 (23 - 59) | | 32 (25 - 42) | 38 (29 - 50) | 9 | 6 | 4 |
| **lower Leg/achilles tendon** | 14 | 2.4 (1.4 - 4.1) | 73 | 4.7 (3.7 - 5.9) | 38 | 3.4 (2.5 - 4.7) | 24 (14 - 40) | 21 (17 - 27) | 23 (17 - 32) | 57 (34 - 96) | | 101 (80 - 127) | 80 (58 - 110) | 11 | 12 | 10 |
| **Elbow** | 11 | 1.9 (1.1 - 3.4) | 14 | 0.9 (0.5 - 1.5) | 24 | 2.2 (1.5 - 3.2) | 23 (13 - 42) | 24 (14 - 41) | 30 (20 - 45) | 44 (24 - 79) | | 22 (13 - 37) | 65 (44 - 97) | 4 | 15 | 9 |
| **Low Back** | 9 | 1.6 (0.8 - 3.0) | 31 | 2.0 (1.4 - 2.8) | 19 | 1.7 (1.1 - 2.4) | 10 (5 - 20) | 10 (7 - 14) | 21 (13 - 33) | 16 (8 - 30) | | 20 (14 - 28) | 36 (23 - 57) | 6 | 6 | 12 |
| **Upper Arm** | 8 | 1.4 (0.7 - 2.8) | 17 | 1.1 (0.7 - 1.8) | 10 | 0.9 (0.5 - 1.7) | **118 (59 - 236)** | 26 (16 - 41) | 83 (45 - 155) | ***163 (81 - 325)** | | 28 (17 - 45) | 76 (41 - 140) | 93 | 8 | 82 |
| **Abdomen** | 4 | 0.7 (0.3 - 1.8) | 6 | 0.4 (0.2 - 0.9) | 16 | 1.4 (0.9 - 2.4) | 8 (3 - 22) | 13 (6 - 29) | 12 (8 - 20) | 6 (2 - 15) | | 5 (2 - 11) | 18 (11 - 29) | 9 | 12 | 6 |
| **Thigh** | 3 | 0.5 (0.2- 1.6) | 3 | 0.2 (0.1 - 0.6) | 3 | 0.3 (0.1 - 0.8) | 60 (19 - 186) | 9 (3 - 29) | 20 (7 - 63) | 31 (10 - 96) | | 2 (1 - 6) | 6 (2 - 17) | 11 | 20 | 28 |
| **Pelvis/sacrum** | 1 | 0.2 (0.0-1.2) | 9 | 0.6 (0.3 - 1.1) | 1 | 0.1 (0.0-0.6) | **106 (15 - 753)** | 28 (15 - 55) | 2 (0 - 14) | ***18 (3 - 130)** | | 16 (9 - 32) | 0 (0 - 1) | 106 | 11 | 2 |

**Supplementary table 6 – Domestic league injury ‘Type’ values**

| **Type** | **Incidence (/1000hrs)** | | | | | | **Mean Severity** | | | **Burden** | | | **Median Severity** | | |
| --- | --- | --- | --- | --- | --- | --- | --- | --- | --- | --- | --- | --- | --- | --- | --- |
|  | **N** | **Artificial** | **N** | **Hybrid** | **N** | **Natural** | **Artificial** | **Hybrid** | **Natural** | **Artificial** | **Hybrid** | **Natural** | **Artificial** | **Hybrid** | **Natural** |
| **Sprain/Ligament injury** | 123 | 21.2 (17.8 - 25.3) | 316 | 20.3 (18.2 - 22.7) | 239 | 21.6 (19.1 - 24.6) | 27 (23 - 32) | 36 (32 - 40) | 31 (28 - 35) | 573 (481 - 684) | 731 (655 - 817) | 675 (595 - 767) | 19 | 19 | 13 |
| **Concussion** | 95 | 16.4 (13.4 - 20.0) | 218 | 14.0 (12.3 - 16.0) | 179 | 16.2 (14.0 - 18.8) | 13 (11 - 16) | 17 (15 - 19) | 14 (12 - 16) | 217 (177 - 265) | 234 (205 - 267) | 224 (193 - 259) | 9 | 8 | 9 |
| **Muscle Strain/Rupture/Cramps** | 82 | 14.1 (11.4 - 17.6) | 183 | 11.8 (10.2 - 13.6) | 127 | 11.5 (9.7 - 13.7) | **31 (25 - 39)** | 26 (22 - 30) | 18 (15 - 22) | ***438 (353 - 544)** | 304 (263 - 351) | 211 (177 - 251) | 21 | 16 | 13 |
| **Haematoma/Contusion** | 62 | 10.7 (8.3 - 13.7) | 170 | 10.9 (9.4 - 12.7) | 138 | 12.5 (10.6 - 14.8) | **13 (10 - 17)** | 8 (7 - 10) | 7 (6 - 8) | 138 (108 - 178) | 89 (77 - 104) | 89 (75 - 105) | 7 | 5 | 5 |
| **Fracture** | 34 | 5.9 (4.2 - 8.2) | 50 | 3.2 (2.4 - 4.2) | 46 | 4.2 (3.1 - 5.6) | 36 (26 - 50) | 43 (32 - 56) | 69 (51 - 92) | 211 (151 - 295) | 137 (104 - 181) | 286 (214 - 381) | 43 | 37 | 48 |
| **Tendon Injury/Rupture/tendonosis/bursitis** | 31 | 5.3 (3.8 - 7.6) | 76 | 4.9 (3.9 - 6.1) | 72 | 6.5 (5.2 - 8.2) | **44 (31 - 62)** | 34 (27 - 43) | 20 (16 - 25) | 233 (164 - 332) | 167 (134 - 210) | 129 (103 - 163) | 13 | 12 | 9 |
| **Other** | 30 | 5.2 (3.6 - 7.4) | 81 | 5.2 (4.2 - 6.5) | 45 | 4.1 (3.0 - 5.5) | **31 (22 - 44)** | 22 (18 - 27) | 17 (13 - 22) | ***160 (112 - 229)** | 113 (91 - 141) | 68 (51 - 91) | 12 | 9 | 6 |
| **Nerve Injury** | 15 | 2.6 (1.6 - 4.3) | 38 | 2.4 (1.8 - 3.4) | 33 | 3.0 (2.1 - 4.2) | 10 (6 - 17) | 18 (13 - 25) | 17 (12 - 24) | 26 (16 - 43) | 45 (33 - 62) | 51 (36 - 72) | 6 | 10 | 6 |
| **Dislocation/Subluxation** | 14 | 2.4 (1.4 - 4.1) | 31 | 2.0 (1.4 - 2.8) | 26 | 2.4 (1.6 - 3.5) | **115 (68 - 194)** | 47 (33 - 66) | 95 (64 - 139) | ***278 (165 - 469)** | 93 (65 - 132) | 223 (151 - 327) | 78 | 27 | 103 |
| **Lesion to cartilage/meniscus/disk** | 13 | 2.2 (1.3 - 3.9) | 31 | 2.0 (1.4 - 2.8) | 23 | 2.1 (1.4 - 3.1) | 79 (46 - 136) | 53 (37 - 76) | 76 (50 - 114) | 177 (103 - 305) | 106 (75 - 151) | 158 (105 - 237) | 33 | 53 | 17 |
| **Laceration/Abrasion** | 4 | 0.7 (0.3 - 1.8) | 10 | 0.6 (0.3 - 1.2) | 9 | 0.8 (0.4 - 1.6) | 5 (2 - 13) | 7 (4 - 13) | 11 (6 - 21) | 3 (1 - 9) | 4 (2 - 8) | 9 (5 - 17) | 5 | 5 | 5 |
| **Other bone injury** | 2 | 0.3 (0.1 - 1.4) | 4 | 0.3 (0.1 - 0.7) | 1 | 0.1 (0.0 - 0.6) | 27 (7 - 108) | 27 (10 - 72) | 117 (17 - 831) | 9 (2 - 37) | 7 (3 - 19) | 11 (1 - 122) | 27 | 32 | 117 |

**Supplementary table 7 – Domestic league injury ‘Match Event’ mean values**

| **Event** | **Incidence (/1000hrs)** | | | | | | **Mean Severity** | | | **Burden** | | | | **Median Severity** | | |
| --- | --- | --- | --- | --- | --- | --- | --- | --- | --- | --- | --- | --- | --- | --- | --- | --- |
|  | **N** | **Artificial** | **N** | **Hybrid** | **N** | **Natural** | **Artificial** | **Hybrid** | **Natural** | | **Artificial** | **Hybrid** | **Natural** | **Artificial** | **Hybrid** | **Natural** |
| **Tackling** | 121 | 20.9 (17.5 - 24.9) | 247 | 15.9 (14.0 - 18.0) | 201 | 18.2 (15.9 - 20.9) | 37 (31 - 45) | 32 (28 - 36) | 30 (26 - 35) | | **777 (650 - 928)** | 505 (446 - 572) | 546 (476 - 627) | 12 | 11 | 12 |
| **Tackled** | 116 | 20.0 (16.7 - 24.0) | 300 | 19.3 (17.2 - 21.6) | 231 | 20.9 (18.4 - 23.8) | 26 (22 - 31) | 32 (28 - 36) | 32 (28 - 36) | | 523 (436 - 627) | 613 (547 - 686) | 664 (584 - 755) | 9 | 12 | 10 |
| **Unknown** | 79 | 13.6 (10.9 - 17.0) | 147 | 9.4 (8.0 - 11.1) | 125 | 11.3 (9.5 - 14.5) | **36 (29 - 45)** | 26 (22 - 30) | 17 (14 - 20) | | **492 (395 - 613)** | 241 (205 - 283) | 187 (157 - 223) | 9 | 10 | 6 |
| **Collisions** | 49 | 8.4 (6.4 - 11.2) | 158 | 10.2 (8.7 - 11.9) | 113 | 10.2 (8.5 - 12.3) | 29 (22 - 39) | 26 (22 - 30) | 21 (18 - 26) | | 246 (186 - 325) | 261 (224 - 305) | 220 (183 - 264) | 13 | 10 | 7 |
| **Running** | 43 | 7.4 (5.5 - 10.0) | 123 | 7.9 (6.6 - 9.4) | 71 | 8.6 (7.0 - 10.5) | 46 (34 - 62) | 41 (35 - 50) | 48 (38 - 60) | | 338 (251 - 456) | 328 (275 - 391) | 308 (244 - 388) | 22 | 18 | 16 |
| **Other** | 35 | 6.0 (4.3 - 8.4) | 46 | 3.0 (2.2 - 4.0) | 35 | 2.8 (1.6 - 3.5) | **40 (29 - 55)** | 13 (10 - 17) | 9 (7 - 13) | | **240 (172 - 334)** | 38 (29 - 51) | 29 (21 - 41) | 13 | 7 | 6 |
| **Ruck** | 34 | 5.9 (4.2 - 8.2) | 117 | 7.5 (6.3 - 9.0) | 95 | 8.6 (7.0 - 10.5) | 35 (25 - 49) | 34 (29 - 41) | 32 (26 - 40) | | 205 (146 - 287) | 258 (215 - 309) | 278 (227 - 340) | 12 | 11 | 11 |
| **Scrum*** | 10 | 3.2 (1.7 - 6.0) | 32 | **10.3 (7.3 - 14.6)** | 31 | 3.7 (2.6 - 5.3) | **84 (45 - 157)** | 41 (29 - 59) | 32 (22 - 45) | | 272 (147 - 506) | **428 (303 - 605)** | 119 (84 - 169) | 32 | 14 | 8 |
| **Lineout** | 9 | 1.6 (0.8 - 3.0) | 20 | 1.3 (0.8 - 2.0) | 10 | 0.9 (0.5 - 1.7) | 19 (10 - 37) | 31 (20 - 48) | 20 (11 - 38) | | 30 (15 - 57) | 39 (26 - 61) | 18 (10 - 34) | 26 | 32 | 15 |
| **Maul** | 6 | 1.0 (0.5 - 2.3) | 24 | 1.5 (1.0 - 2.3) | 26 | 2.4 (1.6 - 3.5) | 11 (5 - 25) | 18 (12 - 28) | 22 (15 - 32) | | 11 (5 - 25) | 28 (19 - 42) | **52 (35 - 75)** | 9 | 9 | 11 |
| **Kicking** | 3 | 0.5 (0.2 - 1.6) | 5 | 0.3 (0.1 - 0.8) | 3 | 0.3 (0.1 - 0.8) | 50 (16 - 156) | 69 (29 - 165) | 17 (6 - 53) | | 26 (8 - 81) | 22 (9 - 53) | 5 (2 - 14) | 27 | 17 | 23 |
